# 2422: Magnetic nanoparticles facilitate tracking of dendritic cells for treatment of malignant brain tumors

**DOI:** 10.1017/cts.2017.48

**Published:** 2018-05-10

**Authors:** Adam Grippin, Elias Sayour, Jon Dobson, Duane A. Mitchell

## Abstract

OBJECTIVES/SPECIFIC AIMS: Immune-based therapies hold great promise for treatment of refractory tumors. However, development is limited by a lack of identified immune correlates to vaccination. We recently showed that dendritic cells (DCs) prolong progression-free survival (PFS) and overall survival (OS) in patients with glioblastoma, and that DC migration to site draining lymph nodes robustly correlates with both PFS and OS. While this appears to be a reliable immune correlate, the complexity of routine labeling for PET and SPECT prohibits validation in a large clinical study. We therefore seek to develop a safe, translatable reporter that can be imaged with a widely available imaging modality. METHODS/STUDY POPULATION: The cationic liposome 1,2-doleoyl-3-trimethylammonium-propane (DOTAP) was loaded with MRI-imageable iron oxide nanoparticles (IONPs) with or without the neutral molecules PEG and cholesterol. The resulting nanoparticles were loaded with RNA to form RNA-NPs that were characterized with transmission electron microscopy (TEM) and used to transfect DCs in vitro; 4.7 T MRI was then used to image particles or cells in agarose gel phantoms. RESULTS/ANTICIPATED RESULTS: TEM images of RNA-NPs indicate the presence of IONP-loaded liposomes. In vitro transfection experiments demonstrate that iron oxide does not reduce RNA-NP-mediated transfection of DCs. Additionally, small amounts of either PEG or cholesterol within RNA-NPs increased transfection of DC2.4s and enhanced T-cell priming by bone marrow-derived dendritic cells. A series of 4.7 T MRI images of particles in cells, spleens, and LNs demonstrated quantifiable differences in particle density between groups. DISCUSSION/SIGNIFICANCE OF IMPACT: This data suggests that IONP-loaded RNA-NPs can be imaged with MRI and manipulated to augment DC function. Future work will include in vivo imaging in mice and safety studies to facilitate translation into first-in-human studies. Successful completion of this project would provide a powerful clinical tool to improve and track patient responses to immune therapy.

